# Effect of Instrument Design and Technique on the Precision and Accuracy of Objective Refraction Measurement

**DOI:** 10.3390/jcm9103061

**Published:** 2020-09-23

**Authors:** Alberto Domínguez-Vicent, Loujain Al-Soboh, Rune Brautaset, Abinaya Priya Venkataraman

**Affiliations:** Section of Eye and Vision, Department of Clinical Neuroscience, Karolinska Institute, 171 77 Stockholm, Sweden; loujain.al-soboh@stud.ki.se (L.A.-S.); rune.brautaset@ki.se (R.B.); abinaya.venkataraman@ki.se (A.P.V.)

**Keywords:** refraction, fogging, monocular/binocular view, precision

## Abstract

Background: To evaluate the precision and accuracy of objective refraction measurement obtained with combinations of instrument design and technique. We also compared the performance of the instruments with subjective refraction measurements. Method and analysis: The objective refraction was measured in 71 subjects with three autorefractometers that have different designs and measurement principles (binocular with fogging, binocular without fogging, and monocular with fogging). Repeatability and reproducibility metrics were calculated for the objective refraction measurements. The agreement of the objective refraction measurements between the three instruments and the agreement with the subjective refraction measurements were evaluated. Results: All three autorefractometers had repeatability and reproducibility limits smaller than 0.70D. The smallest difference (0.10D) in the spherical equivalent was seen between the two binocular instruments. Compared with the subjective refraction, the binocular without fogging technique had the smallest mean difference in spherical equivalent (<0.20D) whereas the binocular fogging technique had the smallest limit of agreement interval (1.00D). For all comparisons, the mean difference and limit of agreement interval for the cylindrical components were lower than 0.10D and 0.75D, respectively. Conclusion: All three instruments evaluated had good repeatability and reproducibility. The binocular fogging technique provided the best agreement with subjective refraction.

## 1. Introduction

Autorefractometers provide fast and accurate starting points for subjective refraction and are extensively used in both clinical practice and research. Refraction without the use of cycloplegic agents to paralyze the ciliary muscles is known to show higher myopic values [1,2,3,4]. From the age of 20 years, this effect was not seen and hence cycloplegic refraction is suggested to be of less clinical relevance [4,5]. There are different autorefractors available, each varying in measurement principle and optical design. Depending on the autorefractometer used, the objective refraction measurements can be performed either with or without fogging, monocular or binocular, through the central or the whole pupil, and with or without open-field viewing. The results of objective refraction measurements vary among autorefractors, and those differences are in most cases clinically significant [6,7,8]. This could be explained due to the measurement principle and design of each.

As new autorefractometers are developed, the reliability of such instruments needs to be assessed. This is done by comparing the objective refraction values obtained from the new instruments with the standard subjective refraction [9]. The subjective refraction is the gold standard method to determine the optical correction needed, as this takes into account both the optical and neural factors. The differences between the objective and subjective refraction values can be due to the instrument design and its measuring principle. For example, proximal myopia can be induced in closed-field autorefractometers [10], and when the built-in fogging system is not used more myopic values can be obtained [5,11].

With continuous development in the measurement principles and designs in the new autorefractors, it is important to assess which combinations provide the most accurate objective refraction values. In the present study, we evaluated the precision and agreement of three autorefractometers that have different measurement principles (monocular/binocular and fogging/no fogging). We also compared the performance of these three instruments with subjective refraction.

## 2. Methods and Materials

### 2.1. Subjects

A total of 71 healthy participants (15 men and 56 women; mean age of 26.6 ± 4.6 years, range 19–40 years) were included in this study. Data from only the right eye per participant were analyzed in order not to artificially reduce the confidence interval around the limits of agreement [12]. This study was approved by the Regional Ethical Committee and adhered to the tenets of the Declaration of Helsinki. The informed consent was obtained from each participant after explaining the purpose, nature, and possible consequences of the study.

The inclusion criteria to participate in this study were no ocular dysfunctions that could affect the refraction, no history of ocular disease or refractive surgery procedure, best corrected visual acuity (BCVA) of 0.0 logMAR or better, intraocular pressure below 21 mmHg, no pregnancy or lactation, and no use of any systemic or ocular medication that could have any impact on the refraction.

### 2.2. Instrumentation

Three different autorefractometers were used in this study: the NVision-K 5001 (Shin-Nippon, Japan), the Eye Refract (Visionix, France), and the WaveAnalyzer 700 (Essilor, France). These instruments, that have different designs and measurement principles, were used to measure the objective refraction of each participant. The main differences among the autorefractometers are summarized in Table 1.

The Eye Refract is a binocular refractor that measures the objective refraction simultaneously on both eyes and provides a semi-open-field view. This instrument combines a digital phoropter with a dual Hartman-Shack sensor and uses fogging while measuring the objective refraction. The Eye Refract has been reported to provide similar refraction and BCVA than a conventional subjective refraction [13]. In this study, the participants were instructed to look at a fixation target displayed on a digital screen at 4.5 m from the instrument.

The NVision-K 5001 is an open-field autorefractor in which the objective refraction is measured monocularly without fogging. This instrument has been reported to provide repeatable [14] and accurate [15] measurements of the refractive error. During the measurement, the participants fixated binocularly to a Maltese Cross placed at 4 m from the instrument.

The WaveAnalyzer 700 combines a Hartmann-Shack sensor, a Scheimpflug camera, and a Placido disc ring to measure objective refraction and anterior segment parameters. This is a closed-field autorefractometer that measures the objective refraction monocularly with fogging technique.

### 2.3. Measurements

All the objective refraction measurements were performed by a single experienced examiner. In total, three objective measurements were performed with each instrument on each participant. The measurements were performed on two consecutive days. On the first day, two measurements were performed in succession with each instrument under repeatability conditions. These measurements were used to calculate the repeatability metrics and assess the agreement among the autorefractometers. On the next day, a third measurement was performed between 23 and 25 h after the last measurement of the previous day. This measurement was used together with the first measurement from day 1 to calculate the reproducibility metrics. On both days, the instrument order was randomized for all participants, and the room illumination was the same.

Another experienced observer measured the subjective refraction on a different day in a subgroup of 40 participants (8 men and 32 women; mean age of 26.3 ± 4.7 years) that were chosen randomly from the study population. The objective refraction measured from the WaveAnalyzer 700 was used as a starting value for the subjective refraction. The subjective refraction was performed with conventional fogging method with the aim of finding the maximum positive/minimum negative spherical value that gives the maximum visual acuity. The cylinder was refined using the Jackson Cross cylinder technique. From the binocular refraction performed, only the values from the right eyes were included in the analysis.

### 2.4. Statistical Analysis

The objective and subjective refractions were measured using the spherocylindrical notation, and were converted into power-vector notation for analysis purposes, using the following Equations [16]:

$$M=S+\frac{C}{2}$$

$$J0=-\frac{C}{2}\cdot \cos2\cdot \alpha$$

$$J45=-\frac{C}{2}\cdot \sin2\cdot \alpha$$

In these equations, *M* represents the spherical equivalent, *J*0 and *J*45 represent the cylindrical vectors, *S*, *C*, and *α* represent the spherical power, the negative value of the cylindrical power, and the cylinder axis, respectively. All statistical calculations were done using the *M*, *J*0, and *J*45.

Descriptive statistics were used to summarize the baseline demographics of the results obtained from each measurement and autorefractor. The precision of each instrument was described in terms of repeatability and reproducibility metrics. For this, the within subject standard deviation (Sw) was calculated using the respective measurements for repeatability and reproducibility. The Sw was estimated from the square root of the residual mean square from the one-way analysis of variance (ANOVA) with the subjects as a factor [12]. The repeatability and reproducibility limits were then calculated as $1.96\cdot \sqrt{2}\cdot S_{w}$ and represent the expected limits that 95% of the measurements should be within. The Pearson correlation coefficient was calculated to determine the relation between the precision of the measurements and M.

A Bland-Altman analysis for repeated measurements was used to assess the agreement among the instruments [17]. The 95% limits of agreement were also calculated. The agreement between the subjective and objective refraction was assessed using a Bland-Altman for non-repeated measurements. An ANOVA was also performed to find whether the differences among the autorefractometers and subjective refraction were statistically significant. In all cases, the statistical significance limit was set to a *p*-value < 0.05.

## 3. Results

### 3.1. Precision

The refractive outcomes obtained with each measurement and autorefractor are summarized in Table 2. On average, the differences among the three measurements taken with the same autorefractor were smaller than 0.10D for M, J0, and J45. These differences were not statistically significant (*p* > 0.05) for any of the components.

**Figure 1:**
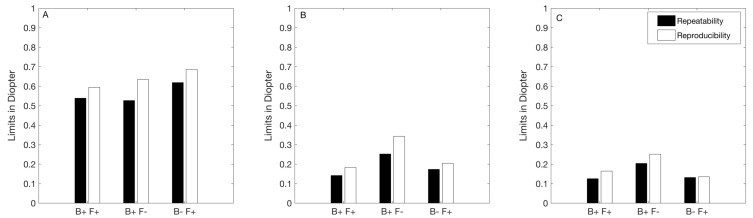
Repeatability and reproducibility limits of objective refraction measurement with the three instruments. The repeatability and reproducibility limits were calculated as $1.96\cdot \sqrt{2}\cdot S_{w}$ where Sw is the within subject standard deviation. (**A**) spherical equivalent, (**B**) cylindrical vector J0, (**C**) cylindrical vectors J45. B+ F+: binocular instrument with fogging, B+ F−: binocular instrument without fogging, and B− F+: monocular instrument with fogging.

Figure 1 shows the repeatability and reproducibility limits of each autorefractor for the M (Figure 1A), J0 (Figure 1B), and J45 (Figure 1C). For all the instruments, the repeatability limits were smaller than the reproducibility limits for each vectorial component. However, the differences never exceeded 0.07D for both fogging instruments. For the B− F+, the maximum difference between the repeatability and reproducibility limits was 0.10D.

The repeatability limits of all instruments for M (Figure 1A) were smaller than 0.65D and similar among each other (differences smaller than 0.10D). For the astigmatic components (Figure 1B,C), the repeatability limits of all the fogging instruments were smaller than 0.25D. The reproducibility limits obtained for M were larger than or equal to 0.60D for the three instruments, and those values were similar among them. For the cylindrical components, the non-fogging instrument showed the largest value for both J0 and J45 (reproducibility limit larger than 0.25D).

**Figure 2:**
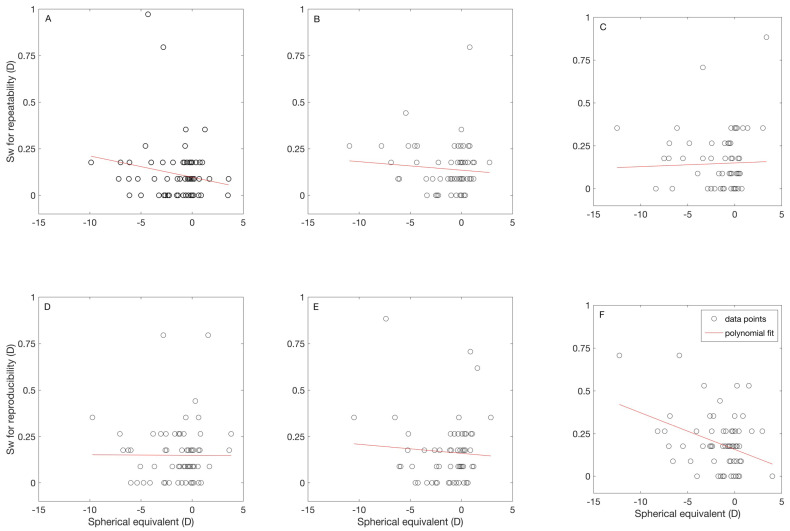
Correlation between spherical equivalent and within subject standard deviation for repeatability (**A**–**C**) and reproducibility (**D**,**E**) for each instrument. The results for the binocular instrument with fogging are shown in (**A**,**D**), for the binocular instrument without fogging are shown in (**B**,**E**), and for the monocular instrument with fogging are shown in (**C**,**F**).

Figure 2 shows the relation between the repeatability (Figure 2A–C) and reproducibility (Figure 2D–F) of the measurements against the M for the three instruments. For B− F+ (Figure 2C,F), the Sw for the reproducibility has a significant negative correlation to M (r = −0.36, *p* = 0.0023). The other parameters did not show a significant correlation to M.

### 3.2. Agreement among Autorefractors

**Figure 3:**
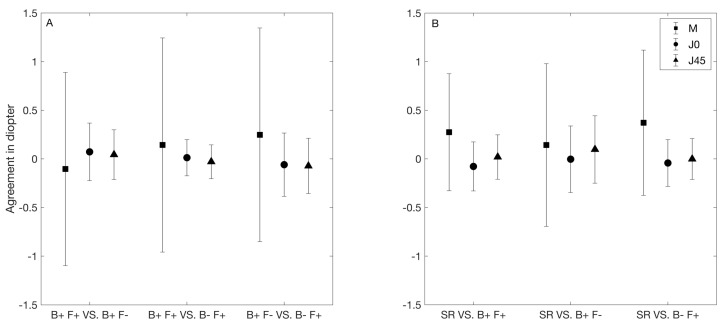
Agreement between different refractive methods. (**A**) Agreement between the objective refraction measurements. (**B**) Agreement between the subjective refraction and the objective refraction measurement by each instrument. The filled symbols denote the mean difference and the error bars denote the 95% agreement limits. M, J0, and J45 represent the spherical equivalent and the two cylindrical vectorial components. B+ F+: binocular instrument with fogging, B+ F−: binocular instrument without fogging, and B− F+: binocular instrument with fogging. SR: subjective refraction.

Figure 3A summarizes the agreement values between the autorefractors. In this figure, the symbols and error bars represent the mean difference and 95% limits of agreement, respectively. On average, the smallest (0.10D) and largest (0.25D) difference in M was seen between the two binocular instruments, and between B+ F− and B− F+, respectively. The limit of agreement interval was about 2.00D for each comparison.

The agreement among the autorefractors was similar for both J0 and J45. The mean difference and limit of agreement interval were always lower than 0.10D and 0.75D, respectively. The lowest limit of agreement interval was obtained between the two fogging instruments (about 0.35D), and that interval was about 0.50 to 0.60D for the other two comparisons.

**Figure 4:**
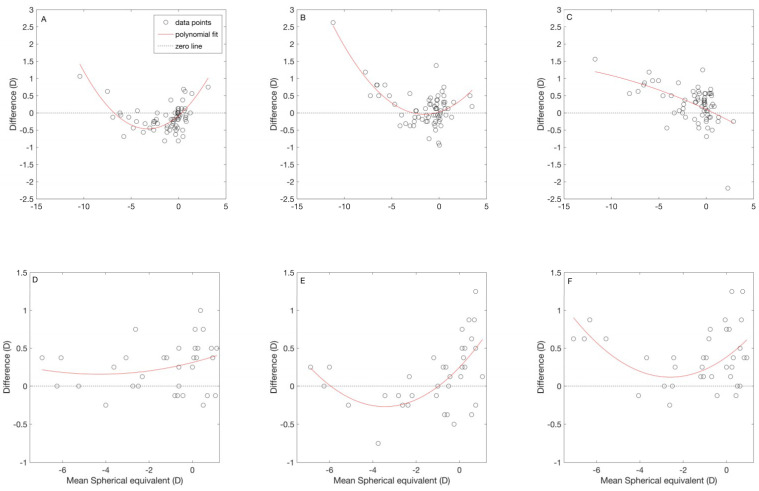
Relation between spherical equivalent and the mean difference among different refractive methods. (**A**–**C**) Relation between the spherical equivalent and the mean difference of each instrument. (**A**) Relation between the binocular instrument with fogging (B+ F+) and the binocular instrument without fogging (B+ F−); (**B**) relation between the B+ F+ and the monocular instrument with fogging (B− F+); (**C**) relation between the B+F− and B−F+. (**D**–**F**): relation between spherical equivalent and the mean difference for the comparison between the subjective refraction and the objective refraction measurement by each instrument. (**D**) relation between the subjective refraction (SR) and the B+ F+; (**E**) relation between the SR and B+ F−; (**F**) relation between the SR and B− F+. For all figures, the open circles denote the mean difference and the red lines represent the quadratic polynomial curve fit.

Figure 4A–C show the relationship between the differences and the mean M for the autorefractor comparisons. The red line represents the quadratic polynomial curve fit of the data. The B+F+ showed a “U” shaped curve with the other two autorefractors. Compared to B+ F+, B+ F− tends to provide less myopic values for mean M between −7.00 D to +0.25 D. Whereas, B− F+ tends to provide more myopic values in general. A trend was seen between the difference and mean M of the comparison of B+F− and B−F+. The B−F+ showed more myopic values as the mean M becomes more myopic.

### 3.3. Agreement between the Subjective and Objective Refraction

Figure 3B summarizes the agreement values between the subjective and the objective refraction measured with each autorefractor. In this figure, the symbols and error bars represent the mean difference and limits of agreement, respectively. On average, the M was positive and lower than 0.50D for each comparison between the subjective and objective refraction. The narrowest and widest limits of agreement interval were about 1.00D (obtained for B+ F+) and 1.75D (obtained for B+ F−).

The agreement between the subjective and each objective refraction was similar for both J0 and J45. For all comparisons, the mean difference and limit of agreement interval were lower than 0.10D and 0.75D, respectively. The narrowest limit of agreement interval was obtained for both B+F+ and B− F+ (about 0.50D in both cases), and the widest interval was obtained for B+ F− (about 0.70D).

Figure 4D–F show the relationship between the differences and the mean M for all three comparisons. The red line in each graph represents the quadratic polynomial curve fit of the data. There is no trend seen for the comparison of subjective refraction with the B+ F+. The B+ F− and B− F+ showed a “U” shaped curve. For the comparison of the subjective refraction and B+ F−, the former provided more myopic values for the mean M between −6.00D and −1.00D. For the comparison of the subjective refraction and B− F+, the entire curve was above zero difference line (dashed line), and the least difference was seen at −2.50D mean M.

## 4. Discussion

We evaluated the precision and agreement of three autorefractometers and compared their performance with the subjective refraction. This was done in order to find the measurement principle and technique (monocular/binocular and fogging/no fogging) that provides the best objective refraction values. All three autorefractometers evaluated had good repeatability and reproducibility parameters. Comparing the objective refraction measurements, the mean difference in M was lower than 0.25D among all the instruments. Compared to the subjective refraction, the limit of agreement intervals were lower than 1.75D for all instruments.

Our precision results showed good repeatability and reproducibility outcomes, and the limits never exceeded 0.70D for M and 0.40D for the cylindrical components. The autorefractometers available for clinical use have been shown to have good repeatability and reproducibility for measuring the objective refraction [7,14,18,19,20]. With the introduction of new instruments, the intra- and inter-session precision needs to be evaluated, although the objective refraction measurements are automated and are seldom influenced by the examiner. There are other factors like patient’s accommodation that can influence the measurements. These could the reason why the precision parameters are worse for M than for the cylindrical components. The repeatability limits were slightly better than the reproducibility limits for all three components measured with each autorefractometer. This could also be due to subject factors such as accommodation and fixation.

The relation between the repeatability and reproducibility of the measurements against M for each autorefractometer (Figure 2) was weak for all instruments, although the correlation for B−F+ was significant (r = −0.36). These results show that the repeatability and reproducibility of these autorefractometers do not depend on the patient’s refractive error. We calculated the measurement tolerance, MT (*MT* = (1.96 · *S_w_*)/*√N*) for N number of measurements [21,22]. For 1, 2, and 3 number of measurements, the MT for M is 0.44D, 0.31D, and 0.25D for measurements taken under repeatability conditions, and 0.49D, 0.34D, and 0.28D for measurements taken under reproducibility conditions. In all cases, one measurement is enough to ensure a MT less than 0.50D.

Comparing the objective refraction measurements among the three different instruments, we found that the limit of agreement interval was about 2.00D. This interval is considerably large clinically and thus these instruments cannot be used interchangeably. Many of the previous studies have also shown similar large limits of agreement intervals [7,10,23,24]. The difference in the objective refraction values obtained with different autorefractometers are suggested to be caused by the fixation target, viewing conditions, fogging system, and wavefront sensors for measurements [23,24].

Comparing the two binocular vision systems used in the present study, the open-view instrument (B+ F−) provided less myopic values than the semi-open-view instrument (B+ F+). Similarly, the B+ F− provided less myopic values than the B− F+ instrument. Though the fogging system is shown to provide less myopic values [5,11], the open/closed view also seem to impact the measurement accuracy. This might be different while measuring in children where accommodation plays a major role [1,4,5]. Comparing the two fogging instruments, the monocular instrument provided more myopic values in general, highlighting the importance of binocular view.

In order to assess the clinical accuracy of the objective refraction measurements, we have to compare it with the subjective refraction. Our results show that on average, the M from all three autorefractometers provide more myopic values than subjective refraction, and the limits of agreement interval was lower than 1.75D. Similar limits of agreement intervals were reported in previous literature using different autorefractometers [13,14,25,26]. Based on the relationship between the differences and the mean M for all three comparisons (Figure 4D–F), the open-view instrument (B+ F−) measured less myopic values compared to the subjective refraction in a range of mean M between −6.00D and −1.00D. This instrument also provided a mean difference close to zero for M (Figure 3B). The other two instruments provided more myopic values in general compared to the subjective refraction.

This also supports the importance of binocular open-view instruments for the measurement of objective refraction. It is essential to know the instrument with the best precision and accuracy in order to optimize the time spent during the subjective refraction, especially in situations where only objective refraction measurements are used and in longitudinal measurements.

The findings from this study cannot be applicable for children since it is known that the accommodation plays a major role in the accuracy of refraction measurements. Our study population had fewer hyperopic subjects and the accuracy in such a case might also be affected due to accommodation in non-presbyopic subjects.

In conclusion, all three autorefractometers had good repeatability and reproducibility parameters. The binocular instruments were more comparable to subjective refraction and the binocular fogging technique had the best agreement.

## Figures and Tables

**Table 1:** Summary of the main differences among the autorefractometers.

Characteristics	Eye Refract	NVision-K 5001	WaveAnalyzer 700
Measurement principle	Wavefront	Retinal image size	Wavefront
Open/Binocular view	Yes	Yes	No
Simultaneous binocular measurement	Yes	No	No
Fogging	Yes	No	Yes
Acronym used in the text	B+ F+	B+ F−	B− F+

B+ F+: binocular with fogging. B+ F-: binocular without fogging. B− F+: monocular with fogging.

**Table 2:** Refractive values obtained with each autorefractor.

	M			J0			J45		
Measurements	1	2	3	1	2	3	1	2	3
**B+ F+**	−1.30 ± 2.44	−1.31 ± 2.47	−1.26 ± 2.45	0.15 ± 0.36	0.16 ± 0.35	0.16 ± 0.36	−0.05 ± 0.19	−0.05 ± 0.20	−0.05 ± 0.18
**B+ F-**	−1.20 ± 2.40	−1.21 ± 2.42	−1.14 ± 2.35	0.08 ± 0.34	0.09 ± 0.35	0.06 ± 0.35	−0.09 ± 0.17	−0.10 ± 0.18	−0.09 ± 0.16
**B− F+**	−1.44 ± 2.73	−1.45 ± 2.64	−1.38 ± 2.62	0.14 ± 0.37	0.15 ± 0.37	0.15 ± 0.36	−0.02 ± 0.20	−0.01 ± 0.21	−0.03 ± 0.21

The values represent average ±1 standard deviation. All values are expressed in dioptres. B+ F+: binocular with fogging. B+ F−: binocular without fogging. B− F+: monocular with fogging. M: spherical equivalent. J0 and J45: cylindrical vectorial components.
